# Hormonal receptors in lung adenocarcinoma: expression and difference in outcome by sex

**DOI:** 10.18632/oncotarget.12244

**Published:** 2016-09-26

**Authors:** Rossana Berardi, Francesca Morgese, Alfredo Santinelli, Azzurra Onofri, Tommasina Biscotti, Alessandro Brunelli, Miriam Caramanti, Agnese Savini, Mariagrazia De Lisa, Zelmira Ballatore, Cecilia Pompili, Michele Salati, Paola Mazzanti, Mariangela Torniai, Stefano Cascinu

**Affiliations:** ^1^ Medical Oncology Unit, Università Politecnica delle Marche, Azienda Ospedaliero-Universitaria Ospedali Riuniti di Ancona, Ancona, Italy; ^2^ Section of Pathological Anatomy and Histopathology, Deparment of Neuroscience, Università Politecnica delle Marche, Azienda Ospedaliero-Universitaria Ospedali Riuniti di Ancona, Ancona, Italy; ^3^ Department of Thoracic Surgery, St. James's University Hospital, Leeds, UK; ^4^ Thoracic Surgery, Azienda Ospedaliero-Universitaria Ospedali Riuniti di Ancona, Ancona, Italy; ^5^ Actual Position: Oncologia Medica-Università degli studi di Modena e Reggio Emilia Modena, Italy

**Keywords:** non-small cell lung cancer, gender differences, androgen receptor, estrogen receptor, progesterone receptor

## Abstract

**Background:**

Lung cancer seems to have different epidemiological, biomolecular and clinical characteristics in females than in males, with a better prognosis for women. The aim of the study is to determine gender differences in lung adenocarcinoma in terms of androgen (AR), estrogen (ER)α and progesterone (PgR) receptors expression and their impact on outcome.

**Results:**

Overall survival was significantly better in ERα and in PgR positive lung adenocarcinoma patients (median survival 45 vs. 19 months).

Eight out of 62 patients showed positive expression of nuclear (n) AR and 18 of cytoplasmic (c) AR with a significantly better survival (49 vs. 19 and 45 vs. 19 months, respectively). There was a significant difference in survival between patients with vs. without c-AR expression (30 vs. 17 months). Finally, in the subgroup of women, median survival was greater in positive expression of c-AR than for women with negative c-AR (45 vs. 21 months).

**Materials and Methods:**

We conducted an analysis on a cohort of 62 patients with advanced NSCLC treated at our institution. We investigated the immunohistochemical expression of n/c AR, ERα and PgR in 62 NSCLC and we correlated it with patients' clinic-pathologic characteristics and with prognosis.

**Conclusions:**

Our results showed that the positive expression of one hormonal receptor could represent a prognostic factor.

Furthermore our study suggests that AR should become object of close examination in a larger series of lung adenocarcinoma patients, also for selection of the patients with best prognosis that can perform more chemotherapy lines.

## INTRODUCTION

Lung malignant neoplasms represent very common tumors and the leading cause of mortality for cancer worldwide [1].

Nowadays, epidemiological data show an exponential increase in its incidence and mortality in women.

Gender discrepancies in lung cancer incidence partially underlay documented differences in tobacco habit. Only in the last years smoke became also a female habit [2] and several studies showed females as more predisposed to smoke tumorigenesis than men [3, 4].

Furthermore lung cancer in women seems to have different characteristics than in men; presenting unfavorable trend considering incidence and survival for females.

The distribution of histological types is significantly different: adenocarcinoma is the principal histotype in women and it is also the most frequent histologic type of NSCLC in non-smokers and young people [5, 6, 7, 8].

Moreover the literature data suggest that females present a greater outcome than men, probably for the influence of female hormones levels on drug pharmacokinetics.

Gender differences in lung disease have proposed a responsibility of estrogens.

One of the greatest biological differences between men and women, indeed, is the presence of female sex hormones and a growing number of studies suggest that estrogens and progesterons, may activate lung carcinogenesis.

**Figure 1:**
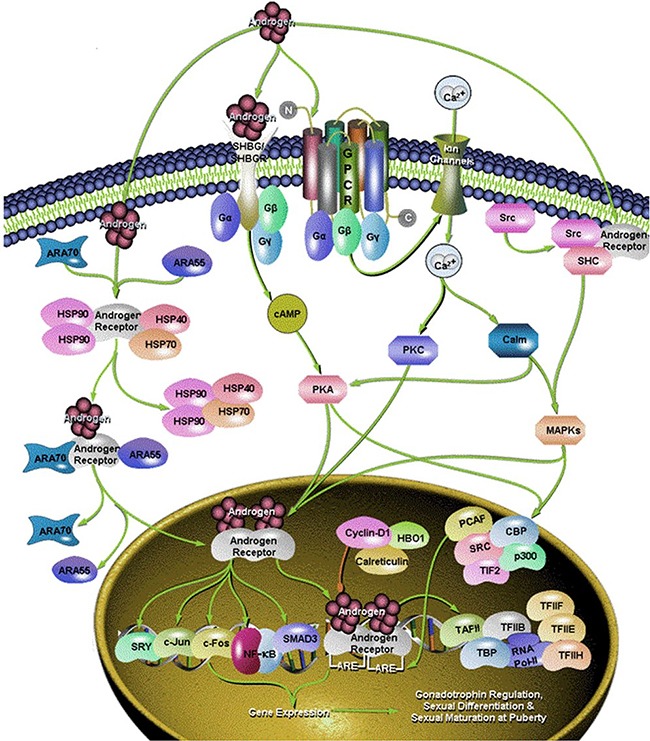
Different signaling pathway of androgen

Some studies on transgenic mice suggest that androgens may contribute, at least in part, to the development and/or progression of lung cancer, too. Androgens may, in fact, enhance the proliferative effect of estrogens [9] (Figure 1).

Nevertheless, an exhaustive analysis of all hormonal receptors has not been conducted, yet.

Our study wanted to establish whether the different hormonal patterns (estrogens, progesterons and androgens receptors expression) have a clinical impact on outcome in lung adenocarcinoma also by sex.

## RESULTS

We conducted an analysis of a cohort of 62 patients (pts) with stage IIIb or IV NSCLC managed at the Department of Medical Oncology at our Institution. The ERα, PgR and n-AR (nuclear androgen receptors) and c-AR (cytoplasmic androgen receptors) expressions were evaluated. Patients' clinical characteristics are summarized in Table 1.

**Table 1:** Clinic-pathological features of 62 patients examined for hormones receptor expression

CLINIC-PATHOLOGICAL FEATURES OF 62 PATIENTS WITH NSCLC	
GENDER	N.
MALES	**36 (58%)**
FEMALES	**26 (42%)**
**AGE**	
MEDIAN [RANGE]	**67,5 MONTHS (34-85)**
**STAGE OF DISEASE AT INITIAL DIAGNOSIS**	
I	**4 (6,4%)**
IIA	**4 (6,4%)**
IIB	**3(4,8%)**
IIIA	**10 (16,1%)**
IIIB	**6 (9,7%)**
IV	**35 (56,5%)**
IIIB/IV	**41 (66,1%)**
**SMOKING HABIT**	
YES	**37 (59,7%)**
NO	**21 (33,9%)**
UNKNOWN	**4 (6,4%)**
**PERFORMANCE STATUS (ECOG)**	
0	**35 (56,5%)**
1	**25 (40,3%)**
2	**2 (3,2%)**
**SITE OF METASTASIS**	
BONE	**22 (35,4%)**
CNS	**9 (14,5%)**
LYMPH-NODES	**8 (12,9%)**
PLEURA	**14 (22,6%)**
LIVER	**6 (9,7%)**
LUNG	**22 (35,4%)**
ADRENAL GLAND	**2 (3,2%)**
OTHER	**412 (66,13,2%)**
**DEATH**	
YES	**44 (71%)**
NO	**18 (29%)**

All women (26%) included in the study were in postmenopausal status.

Median follow up resulted 55.38 months (range 1.02-88.59).

Median OS (mOS) arose 19.5 months (range 0.92 - 89 months) and median PFS reached 9 months (range 1.02 - 68 months).

**Figure 2:**
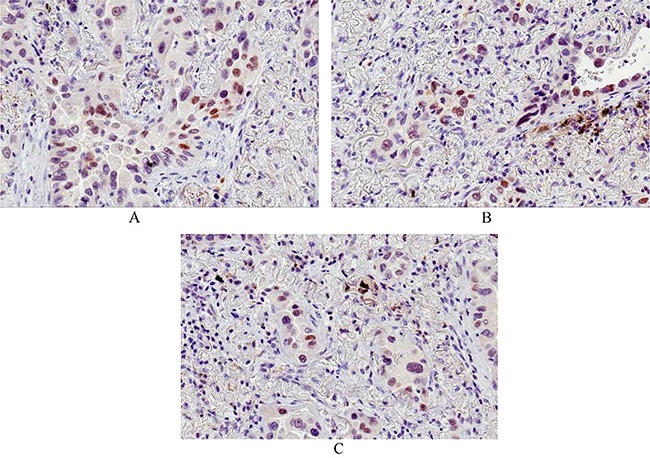
Immunohistochemical staining: ER; PgR; AR **A.** immunohistochemical staining for ER (clone 1D5): nuclear positivity (total magnification 200x) - **B.** immunohistochemical staining for PgR (Clone PgR636): nuclear positivity (total magnification 200x) - **C.** immunohistochemical staining for AR (Clone F39.4.1): nuclear positivity (total magnification 200x).

The results of ERα, PgR and n/c-AR expression analysis are summarized in Tables 2–3 and Figure 2.

**Table 2:** Hormonal receptor expression analysis

	ER		PgR		Nuclear AR		Cytoplasmic AR	
**Receptor expression analysis** **(62 patient in total)**	+	-	+	-	+	-	+	-
	5 (8%)	57 (92%)	4 (6%)	58 (94%)	8 (13%)	8 (13%)	18 (29%)	44 (71%)

**Table 3:** Positive and negative expression of hormones receptors by sex

Sex	ER	PgR	n-AR	c-AR	N. (% By Sex)
Females	Negative	Negative	Negative	Negative	18 (69.3)
Females	Negative	Negative	Negative	Positive	2 (7.7)
Females	Negative	Positive	Negative	Negative	1 (3.8)
Females	Positive	Positive	Negative	Negative	3 (11.6)
Females	Positive	Negative	Negative	Negative	1 (3.8)
Females	Positive	Positive	Positive	Negative	1 (3.8)
Males	Negative	Negative	Negative	Negative	20 (55.6)
Males	Negative	Negative	Negative	Positive	7 (19.4)
Males	Negative	Negative	Positive	Negative	7 (19.4)
Males	Positive	Negative	Negative	Positive	1 (2.8)
Males	Positive	Positive	Positive	Positive	1 (2.8)

The subgroup of patients with n-AR positive expression were all men (7/7=100%), mainly smokers (6/7=85.7%) with stage IV at onset of disease (42.9%) and lung metastasis (42.9%).

The subgroup of patients with c-AR positive expression included mostly men (7/9=77.8%), smokers (7/9=77.8%), with stage IV at onset of disease (6/9=66.7%).

The small subgroup with positive expression of ER and PgR included females (2/2 =100%), with stage II lung adenocarcinoma at diagnosis (2/2=100%) and subsequently developing lymph-node metastasis (2/2 =100%). These data are widely illustrated in Table 4.

**Table 4:** Specific characteristics of patient whit positive hormone receptors

N.	Age	Gender	Smoke	Stage	Site of metastasis	ER	PgR	n-AR	c-AR
1	61	M	Y	IIIB	Unk	0%	0%	2%	0%
2	60	M	Y	IV	Bone	0%	0%	20%	0%
3	62	M	N	IIIA	Lung	0%	0%	20%	0%
4	70	M	Y	IV	Lung	0%	0%	20%	0%
5	76	M	Y	IIA	CNS	0%	0%	20%	0%
6	78	M	Y	IV	Lung	0%	0%	20%	0%
7	82	M	Y	IIIB	Unk	0%	0%	30%	0%
8	68	M	Y	IIIA	Lymph-node; pleura	0%	0%	0%	20%
9	57	M	Y	IV	CNS; lymph-node	0%	0%	0%	40%
10	66	M	N	IV	Bone	0%	0%	0%	40%
11	74	M	Y	IIB	Lung; pleura	0%	0%	0%	50%
12	74	F	N	IV	Lung; bone	0%	0%	0%	50%
13	50	M	Y	IV	Lung; pleura	0%	0%	0%	60%
14	62	M	Y	IV	Lung	0%	0%	0%	70%
15	71	M	Y	UNK	Unk	0%	0%	0%	80%
16	69	F	Y	IV	Liver	0%	0%	0%	85%
17	58	F	N	IV	Pleura; liver; bone	0%	20%	0%	0%
18	53	F	N	I	Unk	2%	9%	0%	0%
19	74	M	Y	IV	CNS	3%	0%	0%	10%
20	76	F	Y	IIA	Lymph-node	10%	8%	0%	0%
21	70	F	N	IIB	Lung; Lymph-node, bone	15%	12%	0%	0%
22	70	M	Y	IIA	Unk	15%	15%	12%	25%
23	62	F	N	IV	Pleura; lung	60%	0%	0%	0%
24	68	F	N	IB	Lung	85%	85%	50%	0%
25	61	M	Y	IV	Lung	0%	0%	0%	0%

OS was significantly better in ER expressed vs. ER negative and in PgR exhibited vs PgR negative lung adenocarcinoma patients (median survival=45 months vs. 19 months in both groups, HR=0.38 [95% confidence interval (CI)=0.16-0.93], *p*=0.03 and HR=0.42 [95% CI=0.15-0.92], *p=* 0.04, respectively).

**Figure 3:**
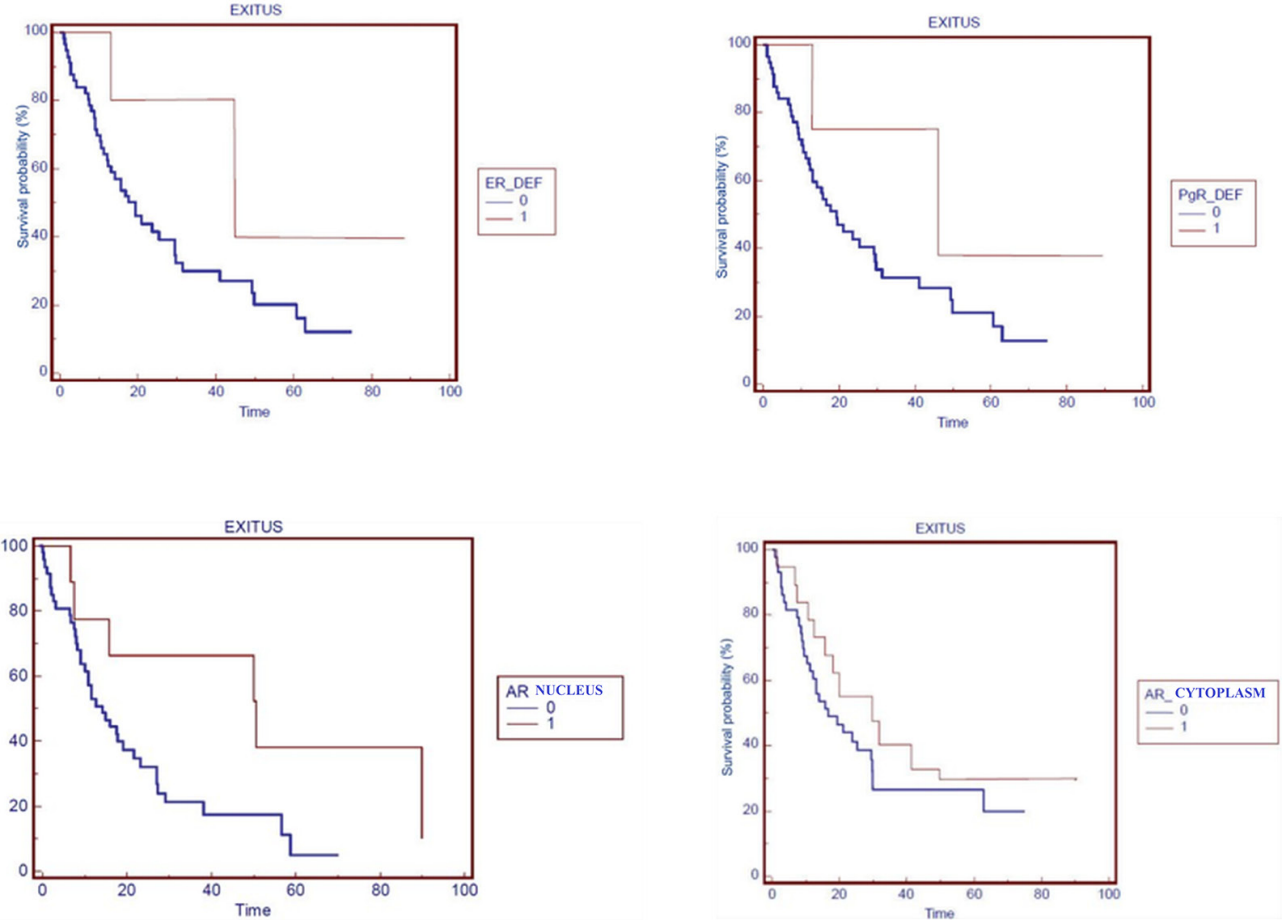
Overall survival by hormones receptors OS in patients stratified by: ERα, PgR, nuclear-AR and cytoplasmic-AR expression (0=negative, 1=positive expression).

Better progression free survival (PFS) in ER and/or PgR positivity patients was showed, although not statistically significant (Figure 3).

n-AR expression was positive in 8 patients (12.9%, 7 males and 1 female) and it was negative in 54 patients (87.1%, 29 males and 25 females).

OS was significantly better in patients with AR expression vs. no AR expression (median survival=49 months in n-AR positive vs. 19 months in n-AR negative lung adenocarcinoma, HR=0.75 [95% CI =0.54-0.96], *p*=0.03) and also in females with AR expression vs. no AR expression (median survival=45 months in n-AR positive vs. 19 months in n-AR negative lung adenocarcinoma, HR=0.31 [95% CI =0.04-0.68], *p*=0.04).

c-AR expression was positive in 18 patients (29.0%, 15 males and 3 females) and it was negative in 44 patients (71.0%, 21 males and 23 females).

Furthermore there was an important difference in OS between positive c-AR expression pts vs. negative ones (30 months vs. 17 months, HR=0.76 [95% CI =0.46-0.95], *p*=0.02).

Finally, in the category of female patients, median survival was greater for those who showed a positive c-AR than for women with negative c-AR (45 months vs. 21 months, HR=0.50 [95% CI =0.12-0.68], *p*=0.03).

A not-statistical significant trend in term of OS was observed in males according to n-AR expression (median survival=49 months in n-AR positive vs. 17 months in n-AR negative lung adenocarcinoma, *p*=0.49) and to c-AR expression (median survival=30 months in c-AR positive vs. 16 months in c-AR negative lung adenocarcinoma, *p*=0.90).

## DISCUSSION

The present study investigates the correlation of hormonal receptors expression with the clinical outcomes of lung adenocarcinoma patients.

In our study we showed a significant survival benefit in patients with positive expression of one of the investigated hormonal receptors (ERα, PgR or AR).

In particular, the expression of ERα, PgR or AR in female patients, resulted in a different prognostic significance according to the specific receptor.. Literature data on ERα, PgR and AR in NSCLC are rather controversial. Several studies primarily focused on the incidence of expression of these receptors and, therefore, on the importance of their role definition as prognostic variables, with different results. Probably these discrepancies are due to a lack of standardization on the method, on the types of antibodies used, on different activation of growth factors and on the interpretation of the same results.

Su and colleagues [10] showed a positive PgR expression with negative ER status in 33% of pulmonary neoplasms collections, with 2% of positivity for both ER and PgR. Conversely, Di Nunno and colleagues [11] didn't discover immunohistochemical reactivity of PgR in 248 lung cancer samples.

Ishibashi *et al.* demonstrated that the expression of PgR, detected in 106 of 228 (45%) NSCLC, is a strong prognostic factor. In particular PgR immunohistochemical positivity was significantly related to sex (*p*=0.0045), and was commonly identified in women. Furthermore PgR immunohistochemical reactivity was meaningfully recognized in adenocarcinoma (*p*=0.0002), appearing inversely related to TNM staging (*p*=0.0085). Finally, PgR expression was correlated with a significantly greater clinical outcome of studied population (*p*< 0.0001).

Our study showed ER and PgR expression in 5/62 patients (8%) and all the three hormonal receptors positive in 2/62 (3%).

The role of progesterone in lung carcinogenesis is unclear. Estrogens and progesterons in vitro synergistically promote angiogenesis and increase tumor progenitor cell compartment as suggested by several studies.

The nuclei of tumor cells showed ERα and ERβ immunohistochemical positivity. In particular estrogen receptor α–positive pulmonary tumors percentage resulted 38%. While, estrogen receptor β–positive lung cancer percentage was 34%.

ERβ status showed a trend of better prognosis, not reaching a statistical significance (*p*=0.1463) [12].

A study by Yan *et al.* analyzed the expression of ERα, as performed in our research, not detecting a significant correlation between ERα positivity and N metastasis but the expression of AR could be associated to progression disease with involvement of N in 105 pulmonary malignancies. The expression of estrogen receptors and androgen ones resulted 14% and 20%, respectively. Positive expression of ER wasn't associated with any clinic-pathological features of studied population. Instead, stage III pulmonary malignancies presented higher rate of androgen receptor positivity than stage I. Furthermore, they observed a significant difference of androgen receptor representation between N0 and N2 stage (*p*=0.0287) [13].

In our study, we showed a meaningful association of ERα expression with a good prognosis. Then the analysis of the expression of the androgens, that it has been poorly considered, also showed a relevant utility in understanding patient outcome.

Rades *et al*. [14] have retrospectively evaluated the expression of ERα, PgR and AR of the 64 patients with NSCLC. The results of this study showed an expression of ERα in 19%, of PgR in 8% and AR in 31% of cases. It was also found that ERα positivity is an adverse prognostic factor in both men and women. In particular supplementary subcategory evaluations showed an inverse relationship between ER-α positivity and locoregional control in females (*p*=0.003) and OS in males (*p*=0.040). The PgR and AR expression did not seem to have a prognostic role. In fact the mOS of the studied population was 26 months. At univariate analysis, ER-α expression appeared inversely related to increased OS (*p*=0.003) but was not associated with PgR (*p*=0.09) or AR expression (*p*=0.64).

Additionally Stabile *et al*. [15] analyzed the expression of ERα, aromatase, ERβ, EGFR and PgR in tumor cells and in normal cells of 183 patients suffering from NSCLC; ERα was significantly expressed in tumor cells, but has not been demonstrated a correlation with prognosis. ERβ, found especially and significantly in lung tumor tissue, was a significant predictor of worse OS. PgR has instead been found most frequently in normal cells than in tumor cells and has been identified as a positive prognostic factor as in our job.

Consistent with other reports, they observed that women had significantly better overall survival (OS) (*p*=0.014; median 4 years) and more extended time to progression (TTP) (*p*=0.009, median 3 years) compared to men (median OS=2 years, median TTP=1 year). ERβ was a significant predictor of worse OS (*p*=0.039; HR=1.05; 95% CI=1.00-1.10) and EGFR approached significance as a predictor for worse OS (*p*=0.060; HR=1.29; 95% CI=0.99-1.68). PgR approached significance as a predictor for longer TTP (*p*=0.066; HR=0.96; 95% CI=0.91-1.00). ERα and aromatase as a single continuous variable showed no effects on survival.

PgR was also stratified with a cut-off of 7 and examined as total staining and by cellular compartment. There were no significant differences in OS, in localization of PgR and in sub-group analysis by sex. However, TTP was significantly longer in patients with high (median TTP=2 years) versus low (median TTP=1 year) PgR total scores (*p*=0.03).

In a retrospective study Novello *et al.* [16] in 130 patients with advanced lung cancer, retrospectively assessed the potential correlation between sex-linked hormone receptor expression and the clinical outcome of patients trated with chemotherapy. The immunohistochemical expression of ER-α, ER-β and PgR, aromatase, epidermal growth factor receptor (EGFR) was assessed. ER-β nuclear expression was higher than ER-α and PgR, whose expression was null or weak (mainly in women). EGFR expression was associated with NSCLC histology, being higher in squamous types and more advanced stage. In men, aromatase positive cases had a worse outcome (*p*=0.03) as well as in men with NSCLC and high ER-β expression. The expression of ERα and PgR was found extremely low and has not reached conclusions on the possible prognostic role and it is restricted to selected subgroups of patients. These data were confirmed in a recent meta-analysis conducted by He *et al* [17, 18, 19, 20, 21, 22].

In addition, the female lung generally presented increased ER-α expression than the male one, as mentioned by Fasco and colleagues [23].

Kawai *et al.* [24] examined ER expression of 132 resected NSCLC specimens using immunohistochemical methods.

ER-α was found in 73% of the tissues examined and its expression decreased the OS (*p*< 0.001).

In order to explain the different expression also in similar studies some authors examined correlation of endogenous/exogenous hormones (menopausal status, hormones replacement therapy etc.) with characteristics of disease (histological grade and stage).

Pesatori *et al.* [25] compared women with diagnosis of pulmonary neoplasms with women healthy volunteers, collecting anamnestic data. The production of females' hormones appeared protective of the carcinogenesis of pulmonary malignancies.

Furthermore Olivo-Marston *et al.* [26] showed that ER-α expression combined with high serum estrogen can predict bad life expectancy in both females and males (*p combined < 0.001*).

A limitation of our study is the limited series of patients and events. Nevertheless, in face of the limitations described, our data show as the study of androgen receptor, less remarkable for the neoplastic pulmonary pathology in literature, can become object of close examination in more numerous series considering the possibility to select the patients with best prognosis that can benefit from more chemotherapy lines. Moreover, the standardization of the methods of ER and PgR analysis also according to the position of the receptors within the cell compartments is warranted, in order to confirm the results and to guide the therapeutic choice even within clinical trials (exogenous hormones, inhibiting hormones, combination of chemotherapy and hormonal therapy).

## MATERIALS AND METHODS

### Patients selection

This study includes consecutive patients with advanced NSCLC treated at the Department of Medical Oncology - Università Politecnica Marche, Italy.

Eligibility criteria included:

- Age >18 years
- Cytological/Histological evidence of lung adenocarcinoma
- Eastern Cooperative Oncology Group (ECOG) performance status 0-2
- Locally advanced (stage IIIB) or metastatic stage (stage IV)

Recorded patient characteristics and clinical features included: age, sex, smoking history, Eastern Cooperative Oncology Group (ECOG) performance status, menopausal status, histological type, clinical and/or pathological stage of disease according to the TNM Seventh Edition (2010), mutational status of EGFR and k-RAS genes, data regarding all the treatments performed by the patients.

Response to therapy was assessed according to the RECIST 1.1 (Response Evaluation Criteria In Solid Tumors) [27] and the therapy toxicity was evaluated using the version 4.0 of “Common Terminology Criteria for Adverse Events” (CTCAE).

### Pathologic analysis

**Figure 4:**
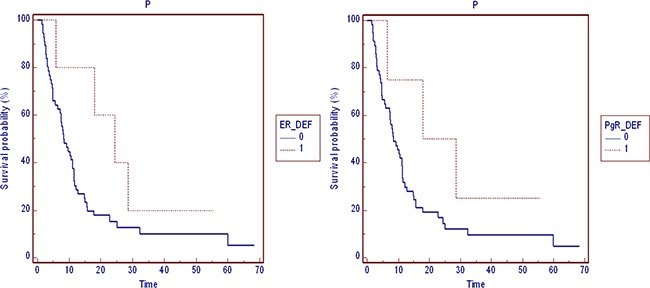
Progression free survival by hormones receptors PFS in patients stratified by: ERα and PgR expression (0=negative, 1=positive expression).

The samples included transbronchial biopsies or pleural resection of the primary lung tumor. The estrogen, progesterone and androgen receptors expression was determined by immunohistochemistry (Figure 4A-4B-4C).

Sections of 3-5 μm thick were deparaffinized and the detection of antigens has occurred in automated manner with DAKO PT Link using ENVISION™ FLEX TARGET RETRIEVAL SOLUTION HIGH pH (50X) for Estrogen receptor (ER) and Androgen receptor (AR), and ENVISION™ FLEX TARGET RETRIEVAL SOLUTION LOW pH (50X) for Progesterone receptor (PgR) (DAKO) at 98°C.

After 70 minutes of treatment, sections were treated with 3% hydrogen peroxide and incubated at room temperature for 30 min with monoclonal antibodies against ER (Clone 1D5) (1:50, DAKO), PgR (Clone 636) (1:50, DAKO) and AR (Clone F39.4.1) (1:60, BioGenex). The staining was completed using ENVISION FLEX ™/HRP (DAKO), as detection system; 3,3-diaminobenzidine-hydrogen peroxide was used as chromogen.

Nuclear immunohistochemical staining was semi-quantitatively assessed by considering the “percentage of positive tumor cells” (range 0-100%), independently from staining intensity.

Immunostaining was assessed by two independent observers who were blinded to the patients' diagnosis.

### Data management and statistical analysis

Primary endpoint of this study was to evaluate the prognostic role of ER, PgR and AR and the correlation between their expression and gender, clinic-pathological features and clinical impact on outcome of patients with advanced lung adenocarcinoma.

OS was defined as the interval between the start of first-line treatment to death or last follow-up visit. The PFS was calculated from the start of treatment until the date of disease progression or death.

Patients who were not reported as died at the time of the analysis were censored at the date they were last known to be alive.

Survival distribution was estimated by the Kaplan-Meier method. Significant differences in probability of surviving between the strata were evaluated by log-rank test. The association between categorical variables was estimated by Chi-square test.

A significant level of 0.05 was chosen to assess the statistical significance.

Statistical analyses were performed using MedCalc version 11.4.4.0 (MedCalc Software, Broekstraat 52, 9030 Mariakerke, Belgium).

### Ethic statement

The research was approved by the ethical committee of our Institution (Comitato Etico Regionale delle Marche, Azienda Ospedaliero-Universitaria Ospedali Riuniti Ancona, Via Conca 71, 60126, Ancona, Italy).

All patients gave their written consent to the study and signed informed consent to all the diagnostic-therapeutic procedures.
